# Fungal foe: exploring cotton’s physiological responses to Verticillium wilt

**DOI:** 10.3389/fpls.2026.1818796

**Published:** 2026-05-13

**Authors:** Xian Yu, Warwick N. Stiller, Warren C. Conaty, Lucy M. Egan

**Affiliations:** 1 CSIRO Agriculture and Food, Narrabri, NSW, Australia; 2 CSIRO Agriculture and Food, Canberra, ACT, Australia

**Keywords:** *Gossypium*, *Verticillium dahliae*, Verticillium wilt, environmental drivers, host plant resistance, integrated disease management, plant-pathogen interaction

## Abstract

Cotton (*Gossypium* spp.) is a globally important cash crop that supports the textile industry and provides valuable by-products such as edible oil and livestock feed. However, cotton productivity and fiber quality are increasingly constrained by Verticillium wilt (VW), a vascular disease caused by the soil-borne fungus *Verticillium dahliae* Kleb. This pathogen can persist in soil for long periods and cause substantial yield and economic losses in cotton worldwide. This review brings together current knowledge of cotton’s physiological responses to VW infection, focusing on how the disease disrupts plant water relations, photosynthesis, nutrient balance, and vascular function. Environmental factors including soil type, temperature, moisture, pH, inoculum density, and nutrient availability, are examined to assess their influence on disease development and severity. The review also explores advances in management strategies such as crop rotation, irrigation, biocontrol, precision agriculture, molecular diagnostics, and breeding for host plant resistance. It emphasizes how insights into cotton’s physiological responses can inform disease management, supporting earlier stress detection and more precise intervention strategies. Furthermore, incorporating physiological monitoring into breeding programs, alongside genomic selection and high-throughput phenotyping, may enhance functional resistance and yield stability under VW pressure. Understanding the complex interactions among the pathogen, host physiology, and environmental conditions is essential for designing proactive, sustainable strategies to mitigate VW impacts and ensure the long-term productivity of the cotton industry.

## 1 Introduction

### 1.1 Overview of global cotton production

The cotton genus (*Gossypium* spp.) includes around 50 species, with 45 diploid species classified into eight genome groups across three main lineages: Australia (C, G, K genomes), the Americas (D genome), and Asia/Africa/Arabia (A, B, E, F genomes) (Stewart, 1994; Percival et al., 1999; Wendel and Grover, 2015; Abdelraheem et al., 2019). The most widely cultivated species, *G. hirsutum* L. (Upland cotton), an AD-genome tetraploid, represents 95% of global cotton production (Wendel and Grover, 2015). Cotton is an important global crop, contributing approximately 35% of the natural fiber used in the textile industry (Khan et al., 2020). Beyond its role in the fiber industry, it is also a key source of edible oil and livestock feed, ranking alongside other leading oilseed crops such as soybean, rapeseed, sunflower, and peanut (Cherry et al., 1978, 1981; Cherry, 1983; Endrizzi et al., 1984; Zhang and Wedegaertner, 2021; Egan et al., 2022; Egan and Stiller, 2022; Man et al., 2022; Zhang et al., 2022a, 2022). Cotton is cultivated in temperate and tropical climates across more than 80 countries. In 2023/24, China remained the world’s largest raw-cotton producer, with an annual output of just over 32 million bales, accounting for around 27% of global production (Meyer and Dew, 2024; The Cotton Wire, 2024). India and the United States follow, and Brazil remains among the top four producers (Meyer and Dew, 2024; The Cotton Wire, 2024). Collectively, these four countries accounted for approximately three-quarters of global cotton output (Meyer and Dew, 2024). In Australia, the 2024/25 cotton crop was estimated to produce at least 4.5 million bales (Cotton Australia, 2025; Meyer and Dew, 2024). Despite being a relatively small cotton producer on a global scale, Australia is one of the top global exporters of raw cotton (Meyer and Dew, 2024). The Australian cotton industry relies on *G. hirsutum* (Stiller and Wilson, 2014), with major production areas in Queensland and New South Wales (Kaur et al., 2019). However, efforts are currently underway to expand cotton cultivation into the tropical regions of northern Australia (Yeates and Bange, 2003; Yeates et al., 2013).

### 1.2 Verticillium wilt in cotton

Disease is one of the most significant biotic stresses limiting cotton production, contributing to reduced yield and fiber quality (Carris et al., 2012; Shaban et al., 2018; Kamburova et al., 2022). VW is a major cotton disease because of its wide distribution and severe impact under favorable conditions (Shaban et al., 2018). VW is caused by the soil-borne ascomycete *Verticillium dahliae* Kleb. The disease was first reported in Virginia in 1914 (Carpenter, 1914). The pathogen has an exceptionally broad host range, affecting more than 400 plant species worldwide (Jian et al., 2004), including major crops such as olives, tomatoes, potatoes, lettuce, and cotton (Bhat and Subbarao, 1999; Inderbitzin et al., 2011).

Numerous studies have demonstrated that environmental conditions strongly influence VW development and epidemic severity. Factors such as heat stress and drought (Zhang et al., 2025b), irrigation (Xiao and Subbarao, 2000), high soil inoculum level (Ashworth et al., 1979), fertilizer application (Albers and Woodward, 2014; Read and Pratt, 2012), light intensity (Pegg and Jonglaekha, 1981), soil pH (Dutta, 1981) and soil type (Land et al., 2017) can all increase the frequency and intensity of VW outbreaks.

A pathotype refers to a genetic or physiological variant of a pathogen that differs in its ability to infect particular host genotypes or to cause specific disease symptoms (Lebeda et al., 2024). In Australia, cotton-growing regions currently recognize two major pathotypes of *V. dahliae*: defoliating (D) and non-defoliating (ND) pathotypes based on their typical symptoms (Le et al., 2020). The D pathotype is generally associated with more severe foliar damage, including chlorosis, necrosis and premature defoliation, and is often considered more aggressive in many international cotton systems. The ND pathotype more often causes wilting, chlorosis, vascular browning, stunting and yield loss with less obvious leaf drop. However, this distinction is not absolute, as symptom severity may vary with host genotype and environmental conditions. In Australia, this is particularly important because the ND pathotype has caused most field-based yield losses and can also show defoliating-like behavior under some conditions (Tzeng and DeVay, 1985; Le et al., 2020; Dadd-Daigle et al., 2021).

### 1.3 Economic and agricultural impacts of Verticillium wilt in different countries

The global spread of VW has caused significant annual losses in cotton production worldwide (Shaban et al., 2018). In the United States, an estimated 480 million bales were lost to VW between 1990 and 2014 (Lawrence et al., 2015). In China, the disease affects more than 40% of cotton-growing areas, causing direct economic losses of approximately USD 250−310 million each year (Wang et al., 2016; Gong et al., 2017). In Australia, VW now occurs across all major cotton-producing regions, although the widespread adoption of resistant cultivars has helped limit its overall impact (Johnson and Nehl, 2004). By contrast, several other cotton-producing countries have reported losses exceeding 50% in severely affected fields (Wu and Subbarao, 2014). Despite generally lower national-level losses in Australia, VW is still considered the most important disease threat to the industry, with yield reductions of 10−62% documented in highly infested fields (Holman et al., 2016).

Developing resistant cotton varieties remains one of the most effective, economical, and sustainable approaches for managing VW, but resistance must be integrated with broader agronomic, environmental, and disease management strategies to achieve consistent control (Zhu et al., 2023; Egan and Stiller, 2022). Although numerous studies have investigated the interaction between *V. dahliae* and cotton, the influence of environmental factors on VW severity in cotton remains poorly understood. Understanding these interactions is crucial, as environmental conditions affect both pathogen activities and host resistance expression.

This review synthesizes current knowledge of VW in cotton, with particular emphasis on how environmental factors, pathogen biology, and host physiological responses interact to influence disease development and severity. It highlights emerging strategies for VW management, including the integration of physiological monitoring, precision agronomy, and breeding approaches that enhance cotton’s resistance to VW infection. By linking environmental, physiological, and genetic perspectives, this review provides a framework for developing more proactive and sustainable management strategies against VW.

### 1.4 Morphological, physiological, and production impacts

Following colonization of the vascular system by *V. dahliae*, cotton develops a range of morphological, physiological, and production-related changes that reduce crop performance. Symptoms may appear in young plants, decline during mid-summer as temperatures rise, and reappear during the fruiting stage (Land et al., 2017). Typical symptoms include stunted growth, reduced lateral branching, chlorosis, wilting and premature defoliation, with reductions in yield, fiber quality and seed quality (Bugbee and Sappenfield, 1970; Wheeler et al., 2012; Xiao and Subbarao, 2000). Defoliation caused by VW further decreases yield by reducing the canopy’s photosynthetic capacity (Land et al., 2017). The timing of symptom onset is critical: early foliar symptoms are associated with greater yield losses, while delayed symptoms result in smaller impacts (Bejarano‐Alcazar et al., 1997). VW also affects important fiber properties, including lint percentage, fiber length, fineness and strength, with the extent of impacts varying among cultivars (Leyendecker, 1950; Pullman and DeVay, 1982; Gutierrez et al., 1983; Tzeng et al., 1985).

A major physiological consequence of VW is disruption to plant water relations. Hyphae and conidia accumulate in the xylem and increase resistance to water flow, creating internal water stress even when soil moisture is adequate (Xiao and Subbarao, 2000). DeVay et al. (1972) showed that wilting in infected cotton is primarily due to impaired hydraulic conductance rather than changes in leaf cell permeability. This hydraulic failure leads to reduced turgor, premature senescence and compensatory responses such as altered stomatal regulation, which further limit carbon assimilation and growth.

VW also compromises photosynthetic processes. Reduced chlorophyll content is one of the earliest physiological signals and becomes more pronounced after prolonged stress (Ji and Peters, 2003; Yang et al., 2022; Lu et al., 2023; Yang et al., 2024). Declines in chlorophyll levels reflect damage to chloroplast structure, disruption of pigment synthesis and restricted CO_2_ diffusion due to stomatal closure. These changes collectively reduce photosynthetic efficiency, accelerate carbohydrate depletion and contribute to reduced biomass accumulation. Because photosynthesis supports fiber development and boll filling, disruptions at this stage directly impact lint yield and fiber quality (Kubicek et al., 2014; Trapero et al., 2018).

Premature senescence caused by VW also impacts cotton yield and fiber quality. VW-induced premature senescence interferes with cellulose deposition and organization during fiber development. This leads to weaker secondary cell walls, a higher proportion of short fibers and reduced fiber strength (Cabrales and Abidi, 2019; Fradin and Thomma, 2006; Chen et al., 2012; Ayele et al., 2020). These structural weaknesses cause complications during ginning and spinning, lowering yarn quality and increasing processing costs. VW-induced production of cell-wall-degrading enzymes and phytotoxins further disrupts fiber formation, exacerbating yield and quality losses.

### 1.5 Upward movement and infection biology of the pathogen

*Verticillium dahliae* survives in the soil for over a decade by forming highly melanized microsclerotia, which allow the fungus to persist through long periods without a suitable host (Davis et al., 1994a; Klosterman et al., 2009). When stimulated by root exudates, these microsclerotia germinate and produce hyphae that grow toward the root surface (Fradin and Thomma, 2006; Klosterman et al., 2009; Zhang et al., 2013; Zhao et al., 2016). The fungus then attaches to the epidermis and forms specialized structures such as hyphopodia, which generate penetration pegs enabling entry through the root cortex (Aguado et al., 2010; Daayf, 2015; Zhu et al., 2023; Eynck et al., 2007; Zhang et al., 2022c).

Two primary explanations have been proposed to describe how *V. dahliae* spreads within the host plant and triggers disease symptoms. The first hypothesis emphasizes the mechanical obstruction of water transport. After entering the root, the fungus reaches the xylem vessels and begins colonizing the vascular system. Only a small proportion of hyphae successfully reach the xylem in the early stage of infection, but once inside, the fungus grows rapidly and produces large numbers of conidia (Fradin and Thomma, 2006; Klosterman et al., 2009; Zhang et al., 2013; Zhao et al., 2016). These conidia move upward with the transpiration stream within the xylem, allowing spread from roots to stems and leaves. Similar upward movement has also been reported in other host species such as olives (Ceccherini et al., 2013) and tobacco (Wright, 1968). Colonization of the xylem by hyphae and conidia interferes with the normal movement of water and nutrients, reduces hydraulic conductance, and creates internal water stress even when soil moisture is adequate. This in turn promotes stomatal closure, reduces internal CO_2_ concentration and photosynthetic activity, contributing to symptom development (Bowden et al., 1990; Haverkort et al., 1990; Bowden and Rouse, 1991; Lorenzini et al., 1997; Saeed et al., 1999; Liu et al., 2018; Zhang et al., 2020).

**Figure 1:**
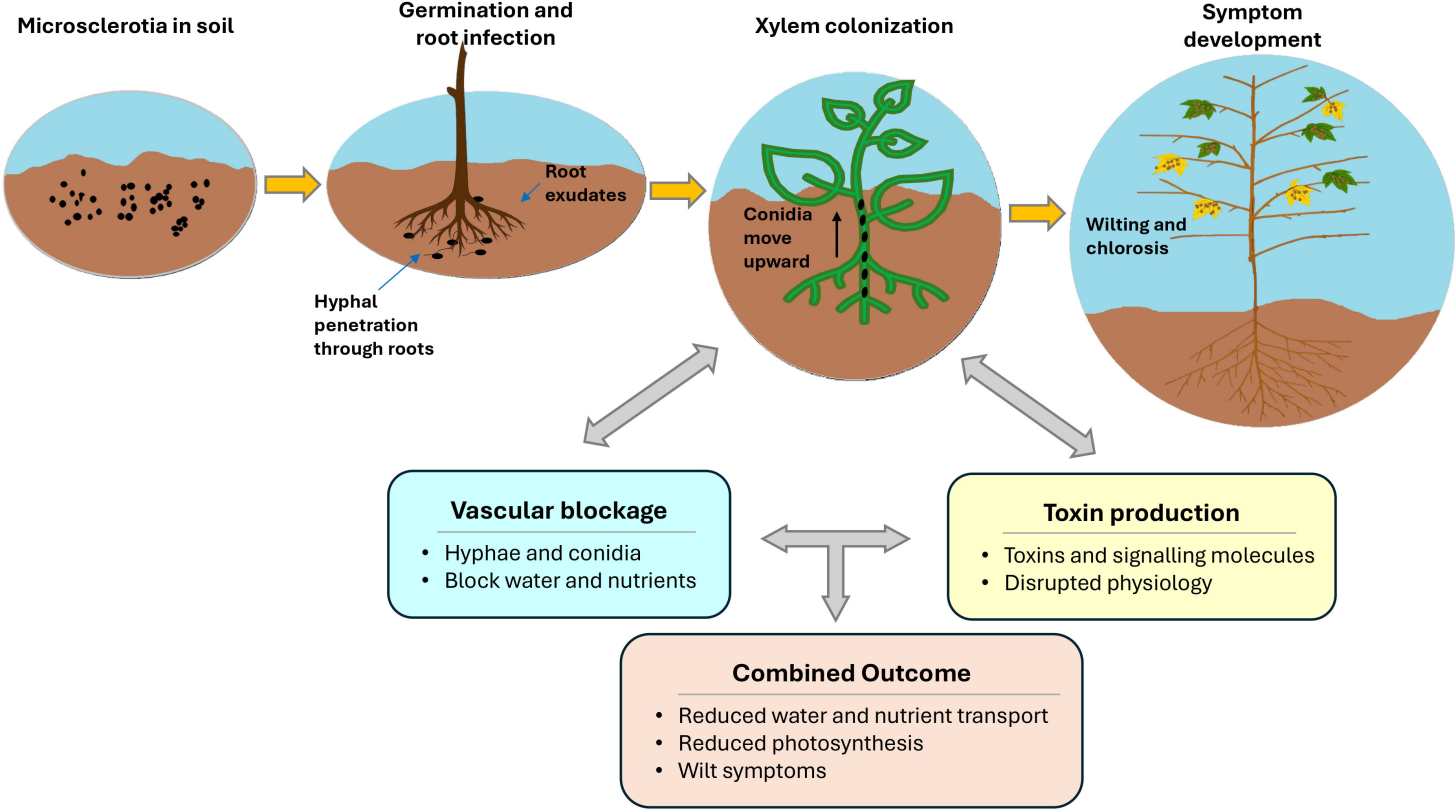
Overview of *V. dahliae* infection and symptom development in cotton.

The second hypothesis highlights the role of toxin-mediated pathogenesis. In addition to vascular colonization, *V. dahliae* produces toxins, effectors and other secondary metabolites that can further disrupt host physiology. These compounds may damage cellular membranes, interfere with normal metabolism, and impair photosynthetic processes, thereby intensifying the physiological effects of infection (Mathre, 1968; Hampton et al., 1990; Lorenzini et al., 1997). In cotton, this idea is supported by the identification of a phytotoxic 18.5 kDa protein from *V. dahliae* culture filtrates that induced leaf dehydration, chlorosis, necrosis and stem discoloration in susceptible seedlings (Palmer et al., 2005), and by evidence that a protein-lipopolysaccharide phytotoxin binds specifically to plasma membranes from cotton seedlings (Meyer and Dubery, 1993). In addition, the secreted effector PevD1 promotes infection by targeting a cotton PR5-like protein, showing that pathogen-derived molecules can interfere with host defense as well as damage host tissues (Zhang et al., 2019). Ethylene is more appropriately interpreted as part of the host response, because *V. dahliae* infection in cotton has been shown to modify ethylene biosynthesis and signaling pathways (Xiong et al., 2020). Current evidence suggests that VW symptoms are best explained by the combined effects of vascular dysfunction and pathogen-induced physiological damage, rather than by mechanical blockage alone (Nachmias et al., 1985; Meyer et al., 1994; Wang et al., 2004; Fradin and Thomma, 2006; Zhang et al., 2020). **Figure 1** summarizes the main stages of *V. dahliae* infection and symptom development in cotton.

This interpretation has practical value for breeding. Resistance traits that reduce root penetration, slow fungal spread in the xylem, or improve compartmentalization of infected vessels may help preserve vascular function. At the same time, traits that improve resistance to fungal metabolites, strengthen oxidative stress responses, or reinforce cell walls may reduce physiological damage. By selecting for genotypes that perform well in both areas, breeders can develop cotton lines with lower symptom severity and more stable performance under VW pressure.

## 2 Environmental factors

**Figure 2:**
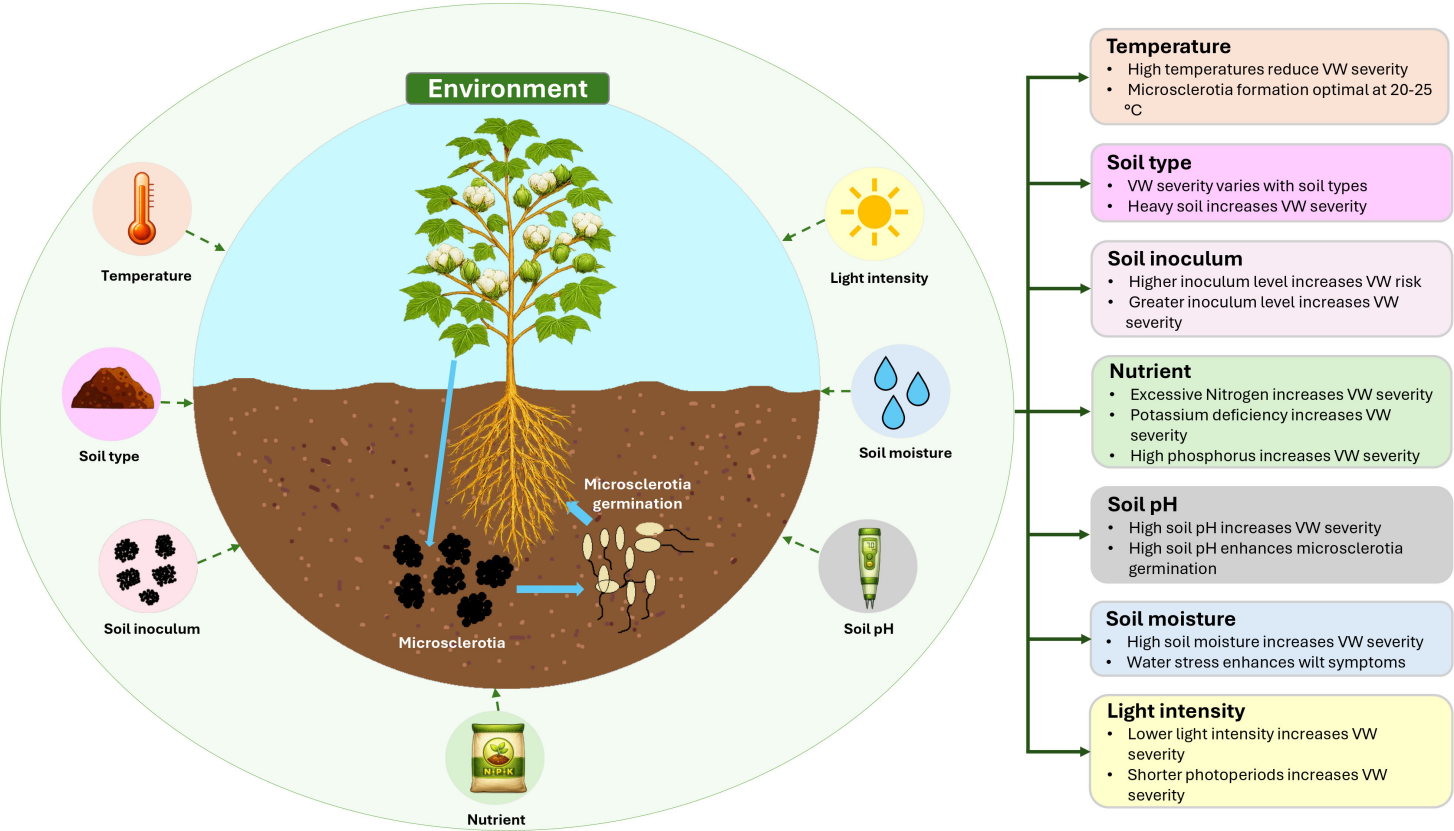
Environmental drivers of Verticillium wilt development in cotton.

Multiple environmental factors influence host infection of cotton by *V. dahliae* (**Figure 2**). Broadly, VW is known to thrive in cool, moist and heavy soils, although the effect of soil type can vary across production systems.

### 2.1 Temperature

Many studies have examined the influence of temperature on cotton infection by *V. dahliae*. These studies provide insights that could aid in timing cotton cultivation to avoid periods when soil conditions are favorable to VW development. However, it is important to recognize that seasonal variation and differences between production areas can create constraints. In some regions, the length of the growing season limits the extent to which producers can align cotton cultivation with more favorable temperature conditions. In field trials, temperatures that were consistently above 28 °C have been found to effectively prevent foliar symptoms and minimize the overall effect of the disease on cotton growth (Garber and Presley, 1971; Pullman and DeVay, 1982).

In culture, temperatures above 30 °C typically inhibit the growth of *V. dahliae*, and prolonged exposure to temperatures above 36 °C is lethal to fungal propagules (Pullman et al., 1981; Wyllie and DeVay, 1970). Temperature can directly or indirectly influence the formation of microsclerotia, often through its effects on plant senescence. Heale and Isaac (1965) identified that the optimal temperature for microsclerotia formation by *V. dahliae* on Czapek-Dox agar (CDA) is 24 °C. Brinkerhoff (1969) found that the ideal temperature range for mycelial growth and microsclerotia development on potato dextrose agar (PDA) is between 22 and 25 °C. Although microsclerotia can continue to form in cotton leaves at temperatures as low as 5 °C and as high as 30 °C, their density significantly decreases at these extremes, and formation ceases entirely at 32 °C (Brinkerhoff, 1969). Hu et al. (2013) further refined these findings, identifying 20 °C as the optimal temperature for microsclerotia formation.

### 2.2 Light

Several studies suggest that VW symptoms become more severe under shorter photoperiods or reduced light intensity (Pegg and Jonglaekha, 1981). In cotton, this relationship is physiologically important because light availability strongly influences stomatal conductance, photosynthesis and carbon assimilation. Under low-light conditions, stomatal opening and photosynthetic activity are reduced, which limits the supply of assimilates needed for growth, defense and recovery from stress. Under VW infection, this effect may become more severe because xylem dysfunction already restricts water movement, promotes stomatal closure and reduces photosynthetic performance (DeVay et al., 1972; Xiao and Subbarao, 2000). As a result, low light may further reduce the ability of infected plants to maintain gas exchange and carbon gain, thereby increasing symptom severity.

In cotton, VW symptoms commonly intensify as plants approach maturity, a developmental stage associated with declining day length but also with increasing physiological demand due to boll load. This heightened sink demand places additional pressure on water, carbon and nutrient allocation, which may further limit the plant’s capacity to tolerate vascular infection and exacerbate symptom expression (Isaac and Harrison, 1968). However, separating the effects of light from those of temperature is challenging in field environments because shorter days typically coincide with cooler conditions that also influence plant physiology and pathogen behavior. Although a few controlled-environment studies have attempted to manipulate light independently, evidence remains limited, especially for cotton. Even so, the consistent trend of higher VW severity under low-light conditions suggests that light should be considered alongside temperature when assessing environmental drivers of disease development. Further work is needed to investigate how light interacts with stomatal regulation, photosynthesis and disease expression under field conditions.

### 2.3 Soil inoculum

The presence of microsclerotia in the soil is essential for disease development. The monocyclic nature of *V. dahliae* means that the amount of microsclerotia largely determines the occurrence and severity of the disease (Ashworth and Zimmerman, 1976; Ashworth et al., 1979; Grogan et al., 1979; Pullman and DeVay, 1982; Nicot and Rouse, 1987; Paplomatas et al., 1992; Harris and Yang, 1996). Studies have shown a direct link between soil inoculum density and the level of vascular browning in cotton stems (Butterfield and DeVay, 1977; Ashworth et al., 1979). Higher inoculum density is generally associated with more severe plant damage. Soil inoculum levels are usually most abundant at planting and tend to decrease as microsclerotia germinate and infect plants, but they rise again after harvest when infected plant material returns to the soil (Evans et al., 1967; Mol et al., 1995; Pegg and Brady, 2002).

### 2.4 Soil pH and soil type

Soil pH and soil type are both key factors influencing cotton productivity and the occurrence and severity of VW. Cotton generally performs best within a pH range of 5.5−8.2, as nutrient availability declines outside this range (CottonInfo, 2015). Soil pH across Australian cotton-growing regions is highly variable. While many coastal and higher-rainfall regions of northern Queensland are characterized by acidic soils, inland cotton-producing areas of northern New South Wales and southern Queensland, particularly those dominated by Vertosols, often have neutral to alkaline soils, with pH values commonly near or above 8.0 (Singh et al., 2003; Wang et al., 1999). Given that much of Australia’s cotton is cultivated on alkaline soils, this factor is particularly relevant. Furthermore, while adjusting soil pH can affect disease development, such management practices are often short-term, costly, and limited in scope (Dutta, 1981).

The development and intensity of VW, seasonal build-up, and the pathogen’s overall distribution appear strongly associated with soil pH levels (Chester, 1942; Young et al., 1959). pH also plays a crucial role during the formation and germination of microsclerotia. Hu et al. (2013) found that the optimal pH for *V. dahliae* microsclerotia formation *in vitro* is 11.5, while Hu et al. (2014) reported that peak germination occurred at pH 8. High pH supports abundant microsclerotia production *in vitro*. Understanding the role of pH in regulating microsclerotia germination under field conditions is important for improving disease management.

Soil type can influence VW expression by affecting moisture retention, aeration and nutrient dynamics, but the relationship is not straightforward. Some studies report higher VW incidence in clay-rich soils with greater silt content, likely due to high moisture retention and reduced aeration that favor infection (Land et al., 2017). In contrast, other studies have found more severe disease in sandy soils, as reported by Rudolph (1929), and Harris and Yang (1996) also observed greater VW severity in lighter, well-drained soils compared with heavier ones. These findings suggest that soil type itself may not have a consistent or direct effect on VW severity. Instead, disease outcomes appear to be driven more by the specific soil properties that influence the plant-pathogen interaction, including water availability, root-zone aeration and nutrient status, together with biological factors such as soil inoculum load, pathogen pathotype and the resistance level of the cotton genotype. This highlights the need to consider underlying soil characteristics rather than soil texture alone when assessing environmental factors that contribute to VW development.

### 2.5 Soil moisture

Soil moisture plays a central role in the disease cycle of *V. dahliae*. Adequate moisture supports microsclerotia germination, conidial movement and root infection, which together increase the likelihood of successful colonization (Arbogast et al., 1999; Land et al., 2017). Once infection occurs, the pathogen restricts water flow in the xylem, making plants more sensitive to water stress even when soil moisture is high (Xiao and Subbarao, 2000). Classic symptoms such as chlorosis, wilting and stunting largely reflect this impaired vascular function, which can eventually lead to reduced yield (DeVay et al., 1972; Duniway, 1973; Tzeng and DeVay, 1985; Tzeng et al., 1985).

Interestingly, rainfed cotton often shows lower VW severity than irrigated cotton, although the underlying mechanisms are not fully clear. Possible explanations include reduced humidity, limited water availability that slows microsclerotia germination, and lower biomass accumulation that reduces the impact of infection (Misaghi et al., 1978). Soil moisture also interacts with soil temperature. Heavy irrigation, especially early in the season, can cool the soil and create more favorable conditions for *V. dahliae*, which grows best at 22−25 °C (Wheeler et al., 2020; Woodward and Wheeler, 2009; El-Zik, 1985). These patterns show that soil moisture does not act alone. Instead, it interacts with other environmental factors such as soil temperature and plant water demand to influence disease development.

### 2.6 Nutrition

Nutrient availability plays an important role in shaping cotton’s physiological responses and susceptibility to *V. dahliae*. Among the major nutrients, nitrogen (N), potassium (K) and phosphorus (P) each influence host-pathogen interactions through different pathways.

Nitrogen is essential for vegetative growth and yield formation, but its relationship with VW severity is complex. While adequate N is needed for healthy canopy development, excessive N has repeatedly been associated with increased VW severity (Albers et al., 2012; Albers and Woodward, 2014; Wheeler et al., 2012). Some studies have reported that low to moderate N levels can delay symptom onset or reduce the incidence of wilt; however, the effects vary across environments and cultivars (Bell, 1992; Albers and Woodward, 2014). These contrasting outcomes suggest that N influences disease development not only through effects on plant growth but also through its impact on physiological status, defense signaling and root-zone conditions.

Potassium is widely recognized as a key element in plant resistance to fungal pathogens due to its role in strengthening cell walls, regulating enzyme activity and maintaining osmotic balance (Read and Pratt, 2012; Ju et al., 2022). In cotton, VW infection has been shown to disrupt K uptake and translocation, resulting in reduced K concentrations in foliage (DeVay et al., 1997). Numerous studies have found that adequate K supply is associated with lower disease severity and greater plant vigor (Ju et al., 2022; DeVay et al., 1997; Amtmann et al., 2008; Huber and Arny, 1985). Increasing K beyond optimal levels, however, does not confer additional resistance (Zörb et al., 2014; Wang et al., 2013; Mengel and Kirkby, 2001). Beyond its direct influence on plant physiology, K also affects the rhizosphere environment by altering soil nutrient dynamics, microbial community composition and root exudate profiles, which may collectively influence *V. dahliae* survival and activity (Kraffczyk et al., 1984).

Phosphorus supports essential metabolic processes, including energy transfer, membrane synthesis and nucleic acid formation (Rausch and Bucher, 2002). Its influence on VW appears to be complex and strongly dependent on P availability. High P supply has been associated with increased disease severity in some studies (Campos and Ortiz, 2020; Tripathi et al., 2022), whereas P deficiency has been shown to activate jasmonic acid-mediated defense responses in cotton, enhancing resistance to *V. dahliae* under controlled conditions (Luo et al., 2021). Plant roots primarily absorb P in the form of orthophosphate through phosphate transporters from the phosphate transporter 1 family (Poirier and Bucher, 2002). However, P deficiency also reduces plant vigor and has been linked to greater VW incidence and yield loss under field conditions (Davis et al., 1994b). These findings indicate that P affects VW severity through a combination of direct effects on defense signaling and indirect effects on overall plant health.

The effects of N, K and P on VW are influenced by their roles in plant growth, structural integrity, defense activation and rhizosphere interactions. No single nutrient acts independently, and their combined influence reflects the balance between promoting healthy growth and maintaining effective physiological and biochemical defenses. Understanding these nutrient-pathogen-plant relationships is essential for interpreting VW patterns across different soils, production systems and environmental conditions.

### 2.7 Summary

Effective management of VW in cotton relies on understanding how different environmental factors interact to shape disease development and severity. Although it is difficult to rank these factors universally across all production systems, temperature and soil moisture appear to be among the most consistently influential drivers because they directly affect microsclerotia germination, pathogen growth, plant water relations, and symptom development. However, their effects are strongly influenced by other factors, including soil inoculum level, soil type, soil pH, light conditions, nutrient availability, and crop growth stage. For example, soil moisture interacts closely with soil temperature and plant water demand, soil type influences disease partly through its effects on moisture retention and aeration, and soil pH may alter both microsclerotia formation and germination. Likewise, nutrient status can affect both plant defense and rhizosphere conditions. These findings indicate that VW severity is determined not by single environmental variables, but by their combined influence on the pathogen, the host, and the environment. Tailored management strategies that reflect these interactions are therefore critical for reducing disease impact and maintaining cotton productivity.

## 3 Verticillium wilt management: impact of host-pathogen interactions

The ongoing challenge of reducing *V. dahliae* levels in soil is compounded by the pathogen’s highly persistent nature (Johnson and Dung, 2010; Dadd-Daigle et al., 2021). A range of management strategies have been used to reduce the impact of VW (Dadd-Daigle et al., 2021), including crop rotation to support disease management on cotton farms (Wheeler et al., 2019) and the development of resistant cotton varieties (Liu et al., 2013). Although chemical control has been explored for VW management, its effectiveness is often limited and inconsistent because *V. dahliae* survives as microsclerotia in soil for long periods and infects plants through the root system (Fradin and Thomma, 2006). These characteristics make it difficult for chemical treatments to reach the pathogen effectively under field conditions (Carroll et al., 2018). In addition, the broad use of chemical control is further limited by variable performance across soils and production systems, as well as by economic and environmental concerns (Kowalska, 2021). For these reasons, chemical control is generally better regarded as a supplementary component of integrated disease management rather than a standalone solution.

**Figure 3:**
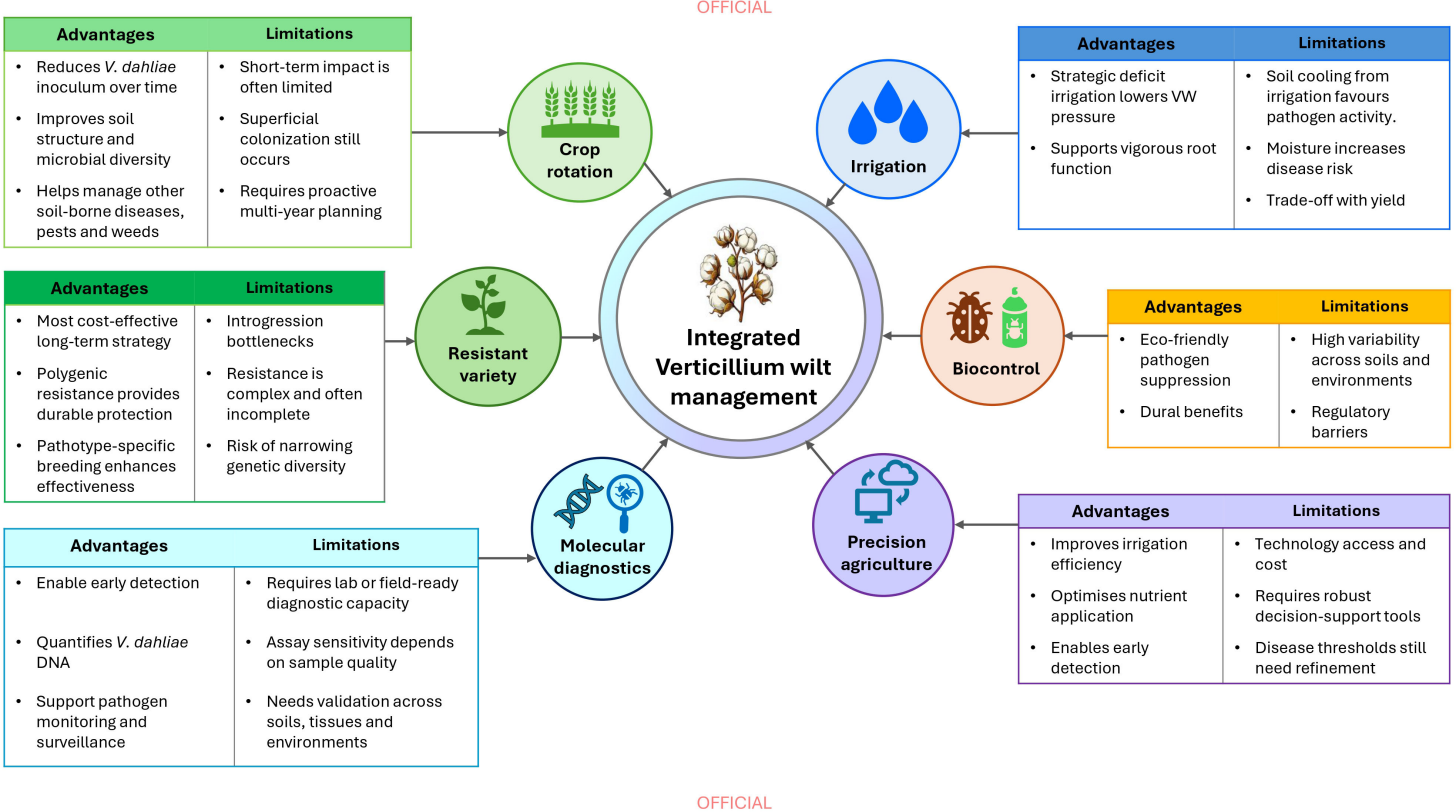
Integrated Verticillium wilt management in cotton.

**Figure 3** summarizes current management strategies and their interactions with environmental variables. Unlike foliar diseases, which can often be controlled through repeated fungicide applications, soil-borne pathogens such as *V. dahliae* reside deep within the soil, which makes traditional chemical treatments less effective. As a result, there remains a significant gap in developing knowledge-based approaches specifically tailored for managing root infections caused by such pathogens (Powelson and Rowe, 1993; Daayf, 2015).

### 3.1 Crop rotation

VW control in cotton requires an integrated strategy, and rotation with non-host crops is a key component (El-Zik, 1985). This practice not only helps manage soil-borne pathogens but also enhances soil structure and promotes beneficial microbial communities associated with plants (Benitez et al., 2017). Typically, crops such as wheat, rice, and maize are rotated with cotton to limit the accumulation of soil-borne inoculum. Crop rotation also plays a crucial role in maintaining soil nutrition, regulating water balance, and controlling other diseases as well as pests and weeds, thereby boosting crop yield (Castellazzi et al., 2008; Chongtham et al., 2017). Nonetheless, the effectiveness of crop rotation can be limited in fields with a long history of VW. Research by Butterfield et al. (1978) and Huisman and Ashworth (1976) highlights that rotation alone may not sufficiently reduce pathogen levels. Huisman and Ashworth (1976) observed that microsclerotia often proliferate after a susceptible crop. Furthermore, Easton et al. (1992) found that a single year of rotation with non-hosts did not significantly lower microsclerotia density in the soil.

Interestingly, non-host crops, despite not exhibiting symptoms of wilt, can still be colonized superficially by *V. dahliae* (Martinson and Horner, 1962; Lacy and Horner, 1966; Malik and Milton, 1980). This superficial colonization is thought to occur because microsclerotia released from infected cotton residues remain in the soil and require time to fully decompose. During this transitional period, the pathogen may colonize the surface of non-host roots without causing disease. However, Wheeler et al. (2012) demonstrated that a well-planned cotton-sorghum rotation strategy over a 10-year period significantly reduced potential damage from VW, with microsclerotia levels being less than 10% of those found in cotton fields. Notably, this rotation was initiated seven years before VW appeared in the field, underscoring the importance of proactive management. Additionally, Xi et al. (2021) reported that a cotton-maize rotation improved soil bacterial diversity and decreased the density of *V. dahliae*, suggesting that such rotations can disrupt pathogen cycles and foster a more resilient soil ecosystem. This evidence supports the idea that while crop rotation is a valuable tool in managing VW, its effectiveness can be influenced by various factors, including the history of the disease in the field and the duration of rotation. Thus, integrating crop rotation with other management practices, such as irrigation, biological control, precision agriculture, molecular diagnostics, and breeding for VW resistance, may be necessary to achieve optimal control of VW.

### 3.2 Irrigation

Irrigation strongly influences the development of VW in cotton because soil moisture directly affects both the pathogen and the plant. Moist soil conditions increase VW incidence, as *V. dahliae* microsclerotia germinate more readily when soils undergo cycles of wetting and drying (Land et al., 2017; Ben-Yephet and Pinkas, 1977). Moisture also supports conidial movement and enhances root infection and colonization (Land et al., 2017). Infected plants often experience heightened water stress because hyphae block the xylem and restrict water transport (Xiao and Subbarao, 2000).

Another important factor is the effect of irrigation on soil temperature. Irrigation events can cool the soil, creating conditions more favorable for *V. dahliae* growth and survival (Karaca et al., 1971). This interaction between soil moisture and temperature helps explain why heavily irrigated fields often show higher levels of disease.

One approach that has shown promise in reducing VW severity is delaying the first post-planting irrigation (Huisman and Grimes, 1989). By postponing early-season watering, microsclerotia are less likely to germinate at a time when the plant is still vulnerable, leading to reduced disease severity and lower yield losses in some studies. However, this strategy also increases the risk of water stress in young plants, which can lead to yield penalties if conditions become too dry (Grimes, 1977). Limiting irrigation more broadly has also been associated with reduced VW development (El-Zik, 1985), but the trade-off between disease suppression and maintaining adequate plant growth remains a major challenge.

Irrigation practices vary widely across cotton-growing regions, and understanding how VW responds to different systems is essential for refining management strategies (Grimes, 1977). Research across multiple crops has shown mixed results: in cotton, higher soil moisture consistently increases wilt severity (El-Zik, 1985), whereas in crops such as maple and mint, VW severity can be greater under true moisture stress rather than excessive moisture (Nelson, 1950; Caroselli, 1957). Karaca et al. (1971) suggested that some of these differences may relate to evaporative cooling following irrigation, which can modify soil temperature and pathogen activity.

Overall, irrigation interacts with several environmental variables, including soil temperature, moisture dynamics and plant water status to shape VW outcomes. Fine-tuning irrigation timing and understanding these interactions at the field scale are therefore critical for improving VW management in cotton.

### 3.3 Biocontrol

Introducing beneficial microorganisms into soils has shown strong potential for controlling soil-borne plant pathogens (Cook, 1993). Biological control methods are valued for their safety and environmental friendliness, as they mitigate the pollution and health risks associated with chemical treatments (Spurrier, 1990; Tjamos et al., 2000). Extensive research has focused on the biocontrol potential of bacterial endophytes against wilt diseases, examining their mechanisms of action and overall efficacy (Eljounaidi et al., 2016). Among these, *Bacillus subtilis* stands out for its dual role in inhibiting plant pathogens and promoting plant growth. This bacterium’s effectiveness has been well-documented in numerous studies (Stein, 2005; Nagórska et al., 2007; Earl et al., 2008). For instance, bio-organic fertilizers containing *B. subtilis* strains HJ5 and DF14 have been shown to significantly reduce the incidence of cotton VW (Zhang et al., 2008; Luo et al., 2010; Lang et al., 2012). Specifically, the strain DF14, in combination with organic fertilizers, demonstrated enhanced efficacy in lowering both disease incidence and fungal counts in the rhizosphere soil (Luo et al., 2010). Importantly, DF14 not only inhibited *V. dahliae* directly, but also changed the rhizosphere microbiome in a way that favored disease suppression, which likely helped reduce overall disease levels (Zhang et al., 2008; Luo et al., 2010). Additionally, fluorescent *Pseudomonas* strains have shown promise as biocontrol agents for *V. dahliae*. These strains have been effective in reducing wilt incidence and severity in a variety of crops, including olive (Mercado-Blanco et al., 2004), eggplant (Malandraki et al., 2008), potato (Uppal et al., 2008) and cotton (Erdogan and Benlioglu, 2010). Notably, Erdogan and Benlioglu (2010) identified fluorescent *Pseudomonas* strains from both weed and cotton rhizospheres, as well as the known strain *Serratia plymuthica* (HRO-C48), as potential biocontrol agents against non-defoliating (ND) *V. dahliae* in cotton.

Although significant progress has been made, large-scale use of biological control strategies still faces several barriers. Scaling up biocontrol methods is difficult because consistent effectiveness across various soil types and environmental conditions is difficult to maintain. Factors such as soil composition, moisture, pH, temperature, and existing microbial communities can affect the survival and activity of biocontrol agents, leading to inconsistent results (Law et al., 2017). Additionally, evenly applying these agents in large agricultural fields poses logistical challenges, as uneven distribution can reduce their effectiveness (Rahman et al., 2024). Developing formulations that keep microorganisms viable during storage, transport, and field application is also crucial for widespread use. Moreover, regulatory requirements add complexity to the commercialization and adoption of biocontrol products (Okoro et al., 2024; Falahzadah et al., 2020). Approval processes can be lengthy, often involving extensive testing to prove safety, effectiveness, and environmental impact. These requirements can delay market entry and raise development costs, creating barriers for smaller companies to introduce new biocontrol products (Falahzadah et al., 2020). The lack of standardized regulations across countries further complicates international trade and broader adoption (Hegde and Vijaykumar, 2025). Addressing these challenges will require advancements in microbial formulation technology, streamlined regulatory processes, and improved strategies for field applications.

### 3.4 Precision agriculture

Effective VW management requires understanding how key farming inputs, particularly soil moisture and nutrients, influence disease development and applying these inputs with precision. Rather than relying solely on technological solutions such as sensors, remote sensing or decision-support platforms, the priority is to manage moisture and nutrient levels in ways that reduce conditions favorable for infection.

Precision irrigation tools such as soil-moisture sensors, drip systems and variable-rate scheduling help maintain soil moisture within the optimal range for cotton, avoiding zones that stay too moist or become waterlogged. These areas tend to support higher pathogen activity. Field studies have shown that variable-rate irrigation systems, including the variable-rate irrigation platform, improve water-use efficiency compared with uniform irrigation and may help reduce VW risk by preventing over-watering in susceptible parts of the field (Vories et al., 2021). Similarly, dynamic management zones updated weekly using drone normalized difference vegetation index (NDVI) and thermal imagery improved water productivity by 12−19% and helped limit persistently moist patches that can encourage disease (Ben-Gal et al., 2025). Variable deficit irrigation has also improved both water and nutrient use efficiency, and by reducing prolonged soil moisture, has helped lower VW pressure in cotton fields (Filintas et al., 2022).

Nutrient management is another area where precision tools support disease control. Variable-rate fertilization guided by canopy sensing (e.g. NDVI, chlorophyll indices) ensures N is supplied according to crop demand, avoiding excess N in high-risk areas (Silvestri et al., 2024). Likewise, soil-nutrient mapping and variable-rate K application help maintain K within the optimal range to support plant defense without unnecessary inputs (Rhymes et al., 2023; Iatrou et al., 2025). Together, these strategies allow growers to balance growth and resistance, reducing VW severity while optimizing nutrient-use efficiency.

Remote sensing and imaging technologies complement these approaches by detecting early signs of VW-related stress such as changes in spectral reflectance or canopy temperature before visual symptoms appear (Tan et al., 2025; Li et al., 2025a; Nie et al., 2024). When combined with soil and weather data in decision-support systems, these tools can guide adaptive irrigation and fertilizer management in real time.

Symptom progression typically begins in the lower canopy, where chlorosis and defoliation develop first, before spreading upward and causing overall canopy thinning (Zhou et al., 2024; Beckman, 1987). However, reliance on visible symptoms alone limits timely disease detection, as physiological disruption often precedes external expression. Recent advances in spectral sensing and chlorophyll fluorescence techniques demonstrate strong potential for identifying Verticillium-induced stress at early stages. Several studies have demonstrated that changes in spectral signatures and fluorescence kinetics can be detected before visible symptoms appear (Yang et al., 2024; Tan et al., 2025; Yang et al., 2022; Li et al., 2025a; Zhang et al., 2025a). These methods provide a foundation for earlier and more precise management interventions for VW.

Future work should continue to define safe thresholds for soil moisture and nutrient levels, test how these thresholds vary across locations, and develop user-friendly decision-support tools for commercial adoption. Integrating agronomic knowledge with precision-agriculture technologies offers a proactive and cost-effective pathway to reducing VW risk while supporting cotton productivity and soil health.

### 3.5 Molecular diagnostics

Molecular diagnostic tools provide an important bridge between disease detection, pathogen monitoring, and practical VW management. Traditional diagnosis based on visible symptoms such as leaf symptoms and vascular browning remains useful, but these symptoms often become apparent only at later growth stages. In contrast, molecular approaches such as PCR, qPCR, LAMP, and newer CRISPR-based detection tools can detect *V. dahliae* more rapidly and sensitively in soil or plant tissues (Tzelepis et al., 2017; Megariti et al., 2024; Wang et al., 2023). These tools are valuable for early detection, monitoring soil inoculum, confirming pathogen presence, and informing management decisions before severe symptoms develop.

Real-time PCR has been used to detect and distinguish *Verticillium* species in soil and plant samples, demonstrating its value for monitoring soil inoculum levels and supporting disease surveillance (Tzelepis et al., 2017). In cotton field research, qPCR-based quantification of *V. dahliae* DNA provides a useful approach for relating pathogen load to disease expression, host response, and environmental conditions (Wang et al., 2022, 2025). This approach is currently being applied in Australian cotton VW field studies to complement visual disease assessment and improve understanding of pathogen-host-environment interactions (Yu et al., unpublished data). More recent developments, including LAMP-CRISPR/Cas12a and quantitative colorimetric LAMP assays, further show the potential for rapid, sensitive, and field-adaptable detection of *V. dahliae* in complex soil or plant samples (Wang et al., 2023; Chen et al., 2024; Fang et al., 2024; Megariti et al., 2024). Integrating molecular diagnostics with traditional disease assessment can improve earlier detection of VW and provide a better understanding of how soil inoculum, host response, and environmental conditions interact in cotton production systems.

### 3.6 Breeding for host plant resistance

Advances in breeding cotton varieties with resistance to VW are crucial for long-term disease management (Egan and Stiller, 2022). Efforts to identify sources of disease resistance have improved understanding of VW resistance (Colella et al., 2008; Gossen and Jefferson, 2004; de Miranda et al., 2010). Among the four cultivated cotton species, *Gossypium barbadense* exhibits greater resistance to VW. However, attempts to transfer these resistant traits into commercial Upland cotton (*G. hirsutum*) have largely been unsuccessful (Wilhelm et al., 1974). This reflects the genetic complexity of VW resistance and the potential trade-offs with agronomic traits such as yield and fiber quality. Overcoming these constraints will require further research into mechanisms and breeding strategies that improve disease resistance without compromising commercial performance.

While genetic markers associated with resistance have been identified, significant challenges remain (Bolek et al., 2005; Wang et al., 2007, 2008; Zhang et al., 2014; Baytar et al., 2017; Bardak et al., 2021; Wilson et al., 2024). For instance, resistance is often polygenic, meaning that multiple genes contribute to cotton’s ability to resist VW. This makes the breeding process slow and resource intensive. Moreover, resistance in one variety may not be durable across different environments due to the pathogen’s capacity to adapt and evolve, raising concerns that resistance may be less durable in some systems. Although this is a long-term concern, there has been no evidence in Australia that the pathogen is overcoming HPR. Rather, the farming system appears to encourage pathogen prevalence and proliferation.

Because populations of *V. dahliae* vary by region and may carry different pathotype profiles, breeding for pathotype-specific resistance is the immediate goal for many programs. For example, Wilson et al. (2024) examined resistance to VW in the ND strain of *V. dahliae* using 240 F_7_ inbred lines from a cross between a resistant variety (MCU-5) and a susceptible variety (Siokra 1-4). Their findings indicated that resistance measured by plant survival and shoot growth was complex but primarily associated with chromosomes D03 and D09. Similarly, Göre et al. (2017) evaluated seedling resistance to both D and ND pathotypes in cotton crosses between Giza 45, an Egyptian extra-long-staple (*G. barbadense*) variety with moderate vascular disease resistance, and Tex-Cotton 3, a Texas-bred Upland (*G. hirsutum*) line with partial VW resistance. They found that resistance in both cultivars was governed by two recessive genes without maternal influence and recommended the use of large populations to improve gene transfer efficiency and fixation of these resistance loci.

Ultimately, stacking multiple resistance genes or well-defined genomic regions targeting different *V. dahliae* pathotypes offers a promising pathway for developing broader and more durable resistance. However, successful gene stacking depends on accurately identifying the key loci and understanding how they interact across environments, which remains an ongoing challenge for cotton. Overreliance on a narrow genetic base for resistance can also reduce overall genetic diversity in breeding populations, potentially slowing future progress in traits such as yield potential, fiber quality and stress tolerance. For these reasons, integrating HPR with diverse germplasm, clearly characterized resistance regions and complementary management practices will be essential for sustainable long-term control of VW.

## 4 Future directions: integrating cotton physiological monitoring into Verticillium wilt management

**Figure 4:**
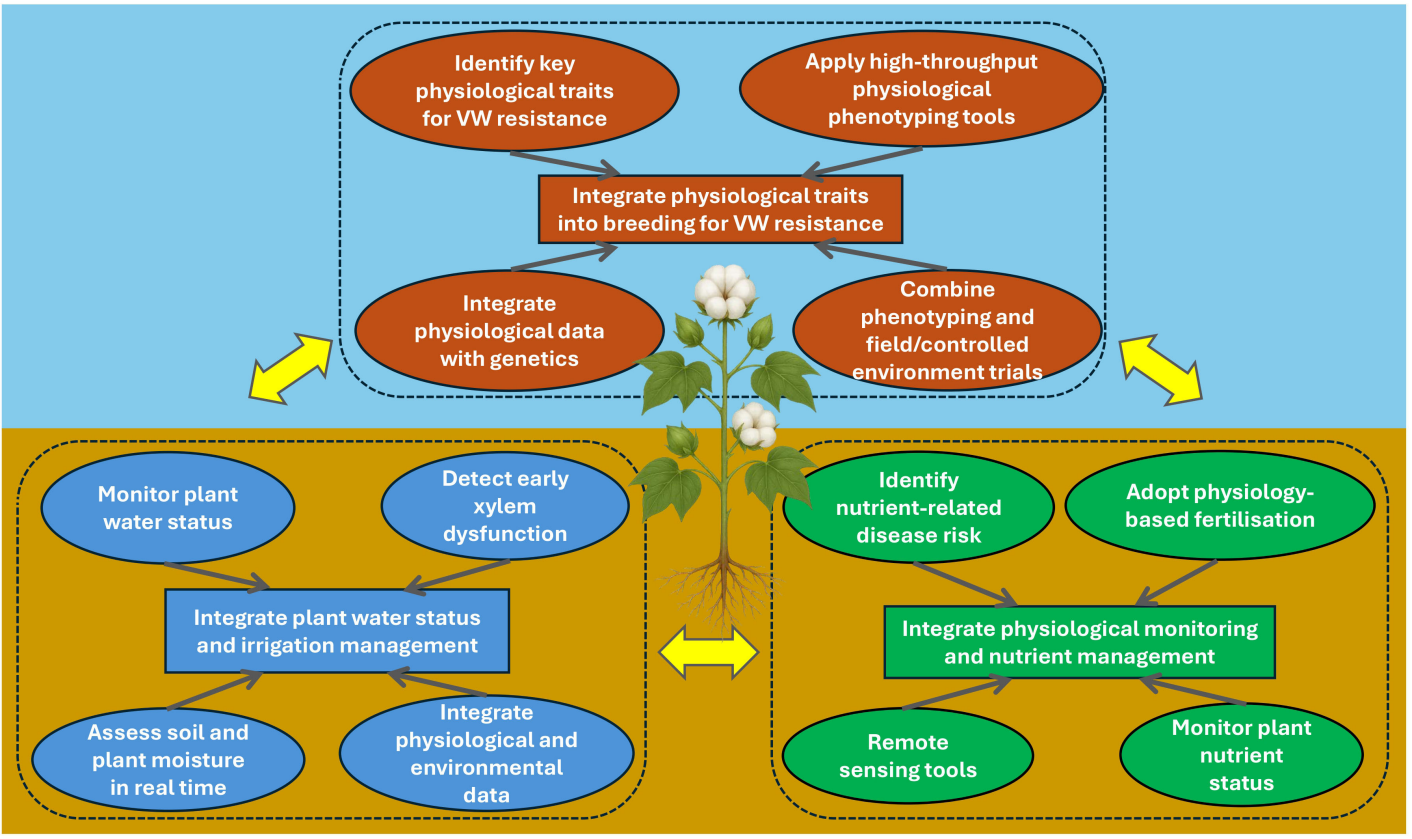
Integration of physiological monitoring into Verticillium wilt management strategies in cotton.

Current management strategies for VW in cotton, such as crop rotation, irrigation management, biocontrol, precision agriculture, molecular diagnostics, and HPR, vary widely in their application and effectiveness. However, Many of these approaches focus on reducing pathogen pressure or improving crop management, while physiological monitoring provides an additional pathway for understanding cotton’s functional response to infection. An emerging research priority is the integration of physiological monitoring into breeding programs and VW management, alongside genomic selection and high-throughput phenotyping (**Figure 4**). By monitoring the crop’s physiological status, it becomes possible to detect early stress responses, implement timely interventions, and enhance yield stability under VW pressure.

### 4.1 Integrating plant water status and irrigation management

*V. dahliae* infection disrupts xylem function, reducing water transport and thereby impairing photosynthesis, with subsequent reductions in both yield and fiber quality (Bugbee and Sappenfield, 1970). Monitoring plant water status, for example through measurements of leaf water potential, canopy temperature or transpiration rates, can provide early indicators of xylem dysfunction. Studies in various crops have demonstrated that pathogen-induced reductions in hydraulic conductivity are closely related to decreased transpiration and stomatal conductance, reflecting early impairment of xylem flow (McElrone et al., 2003; Paljakka et al., 2020; Li et al., 2025b). Similarly, thermal imaging studies have shown that canopy temperature rises as vascular wilt progresses, owing to restricted water movement and reduced evaporative cooling from the canopy (Calderón et al., 2013, 2015; Pineda et al., 2020). More broadly, a recent review on plant hydraulics highlights that these parameters provide reliable, integrative indicators of vascular integrity and can be used to detect early hydraulic failure under disease stress (Torres-Ruiz et al., 2024).

Building on this understanding, integrating physiological monitoring with irrigation management offers a promising direction for VW control in cotton. Real-time assessment of soil moisture and plant water status can guide irrigation (Christenson et al., 2024), maintaining optimal water availability that sustains plant function while avoiding excess moisture conditions conducive to *V. dahliae* germination and infection (CottonInfo, 2022). Canopy temperature measurements, in particular, can signal emerging water stress or xylem dysfunction, enabling timely adjustment of irrigation timing and volume (Wanjura and Upchurch, 2000). By linking physiological and environmental data through precision agriculture, water use can be optimized to improve cotton’s resistance to infection while minimizing pathogen proliferation (University of Southern Queensland, 2023). This integration represents a shift toward proactive, physiology-based VW management that balances disease suppression with sustainable resource use.

### 4.2 Integrating physiological monitoring and nutrient management

Nutrient management is another important area where physiological monitoring can enhance VW management in cotton. Nutrient imbalances, particularly excessive N or insufficient K, have been linked to increased disease susceptibility and greater symptom severity, as they influence plant vigor, tissue composition, and defense activation (Huber and Watson, 1974; Albers and Woodward, 2014; Ashworth et al., 1985). A physiology-based approach, using variable-rate fertilization guided by real-time assessments of plant tissue nutrient status, enables more accurate nutrient delivery aligned with plant demand. Recent advances in remote sensing, including hyperspectral and multispectral imaging, allow spatial mapping of canopy N and K status, supporting fertilizer application that maintains optimal nutrition without promoting excessive vegetative growth (Misbah et al., 2021; Zheng et al., 2022). Such targeted management improves nutrient-use efficiency and reduces the risk of stimulating soft, nutrient-rich tissue favorable to disease development. Continuous assessment through leaf tissue analysis (Radrizzani et al., 2011), sap testing (Lu et al., 2022), and canopy reflectance indices (Xue and Su, 2017) can identify zones of nutrient limitation or imbalance before visible stress occurs. By combining these physiological insights with precision nutrient-delivery systems, growers can implement adaptive strategies that sustain plant health, maintain tissue integrity, and improve the plant’s capacity to withstand infection. This integrated, physiology-based approach to nutrient management therefore provides a proactive tool for managing VW in cotton.

### 4.3 Integrating physiological traits into breeding for VW resistance

Physiological monitoring provides a powerful conceptual framework for breeding aimed at enhancing VW resistance in cotton. While traditional breeding has largely focused on identifying pathogen-specific resistance genes or quantitative trait loci, incorporating physiological traits into selection criteria offers a pathway toward functional resistance that extends beyond single genetic effects. However, many physiologically relevant traits are multigenic, environmentally sensitive and difficult to select for directly, and progress in breeding for such traits has therefore been slow and challenging in practice.

Despite these limitations, several traits remain biologically important targets for improving VW resistance. These include the ability to maintain photosynthesis under VW infection (Ayele et al., 2020), favorable root system architecture such as thicker roots and fewer fine lateral branches that may reduce infection sites (Cardoni et al., 2021; Kuijken et al., 2015), efficient water and nutrient use (Ayyaz et al., 2025), and the maintenance of vascular function and structure under VW infection (Ayyaz et al., 2025; Ayele et al., 2020). These traits influence disease resistance and yield stability, and while their routine deployment in breeding programs may be difficult in the short term, they provide valuable guidance for long-term improvement and for integrating physiological phenotyping with genomic selection and controlled-environment screening.

Advances in high-throughput physiological phenotyping now enable systematic assessment of these traits (Jangra et al., 2021; Gill et al., 2022; Fu et al., 2022). Chlorophyll fluorescence imaging can quantify photosynthetic efficiency and detect early stress responses (Yang et al., 2024), thermal imaging provides insights into transpiration dynamics and stomatal regulation (Calderón et al., 2013; Pignon et al., 2021; Pineda et al., 2020), and root scanning or X-ray tomography allows detailed evaluation of root system architecture and vascular integrity under pathogen pressure (Teramoto et al., 2020; Hou et al., 2022; Ingel et al., 2022). Combining these tools with controlled VW inoculation or field trials allows for precise phenotyping of germplasm under realistic disease scenarios.

Integrating physiological traits with genomic information through approaches such as genome-wide association studies (Susmitha et al., 2023), genomic selection (Escamilla et al., 2025) and marker-assisted selection (Mores et al., 2021) can accelerate breeding for disease resistance. Breeders can identify and select genotypes that perform well physiologically under VW stress by linking functional traits, such as photosynthetic capacity, root architecture, water and nutrient use, and vascular integrity to genetic markers (Poland and Rutkoski, 2016). This approach enables the development of cotton varieties that perform better under the combined pressures of pathogen infection and variable environmental conditions. By integrating physiological phenotyping with advanced genomic and breeding technologies, future research can develop cultivars that not only exhibit strong disease resistance but also maintain productivity and fiber quality under stress. This integrated approach provides a proactive and sustainable pathway for managing VW while enhancing overall crop resistance and performance.

## 5 Conclusion

Verticillium wilt remains a major constraint to cotton productivity because it arises from interactions among host physiology, pathogen biology, and environmental conditions. Infection by *V. dahliae* disrupts water transport, photosynthesis and nutrient balance, leading to substantial yield loss when conditions favor pathogen activity. Environmental drivers such as soil moisture, temperature and nutrient status strongly influence both pathogen behavior and the plant’s ability to maintain defense.

A key insight from the Australian context is that there is currently no evidence that *V. dahliae* is overcoming host plant resistance. Instead, farming-system factors including persistent soil moisture, nutrient imbalances and inoculum build-up appear to be the dominant contributors to disease expression. This highlights the need for integrated management that combines host resistance with improved irrigation, nutrient management and rotation practices.

Long-term progress will depend on linking physiological understanding with genomics, breeding and precision agriculture tools. Identifying key resistance loci, refining gene-stack strategies and improving early-stress detection will support more stable resistance across environments. Together, these advances offer a pathway to more predictable and sustainable management of VW in cotton.
